# A Case of JAK2V617F-Negative Myeloproliferative Neoplasm in a Young Female Presenting With Extreme Thrombocytosis

**DOI:** 10.7759/cureus.50679

**Published:** 2023-12-17

**Authors:** Kelash Kumar, Assile Koubeissy, Arichanah Pulenthiran, Amrat Kumar, Amit Gulati, Brian Wolf, Stephen Peeke

**Affiliations:** 1 Department of Internal Medicine, Maimonides Medical Center, Brooklyn, USA; 2 Department of Internal Medicine, Bassett Medical Center, Cooperstown, USA; 3 Department of Hematology and Oncology, Maimonides Medical Center, Brooklyn, USA

**Keywords:** hydroxyurea use, splenomegaly, jak inhibitor, extreme thrombocytosis, chronic myeloid leukemia (cml)

## Abstract

Thrombocytosis is a commonly observed condition in clinical practice and typically results from various pathophysiological factors, such as iron deficiency, blood loss, infection, medications, rheumatologic conditions, malignancy, asplenia, post-splenectomy, or familial factors. However, extreme thrombocytosis, defined as a platelet count > 10,000 K/UL (equal or greater than a million), is a rare occurrence. In this report, we present a compelling case of severe thrombocytosis attributed to underlying chronic myelogenous leukemia (CML), further complicated by coexisting iron deficiency. It is essential to emphasize that not all instances of extreme thrombocytosis are indicative of essential thrombocythemia. Hence, maintaining a high level of suspicion for non-ET myeloproliferative neoplasms (MPNs) such as CML, as well as other underlying conditions like iron deficiency anemia, is crucial for accurate diagnosis and timely management.

## Introduction

Chronic myelogenous leukemia (CML), alternatively referred to as chronic myelocytic, chronic myelogenous, or chronic granulocytic leukemia, is a condition characterized by the abnormal and uncontrolled growth of granulocytic cells while still preserving their capacity to differentiate. This disorder is characterized by the dysregulated production and uncontrolled proliferation of mature and maturing granulocytes, displaying mostly normal differentiation. Consequently, there is an excessive presence of granulocytes and their immature precursors, occasionally accompanied by blast cells. The defining genetic abnormality is the translocation of t(9;22)(q34;q11.2), leading to the formation of the BCR/ABL1 fusion gene, commonly referred to as the Philadelphia chromosome [1]. CML constitutes 20% of all adult leukemia cases. Approximately 40% of CML patients display no symptoms, and diagnosis relies solely on laboratory findings [2]. Typically, CML patients exhibit leukocytosis, but thrombocytosis is a rare presentation [3]. In instances of extreme thrombocytosis, defined as a platelet count >10,000 K/UL, essential thrombocytosis is the more common diagnosis, with up to 50% of cases involving a JAK2V617F mutation [4], followed by calreticulin (CALR) and myeloproliferative leukemia (MPL) mutations. In this report, we present a case of CML where marked thrombocytosis with accompanying leukocytosis was observed, and interestingly, no JAK2V617F mutation was found.

## Case presentation

**Figure 1:**
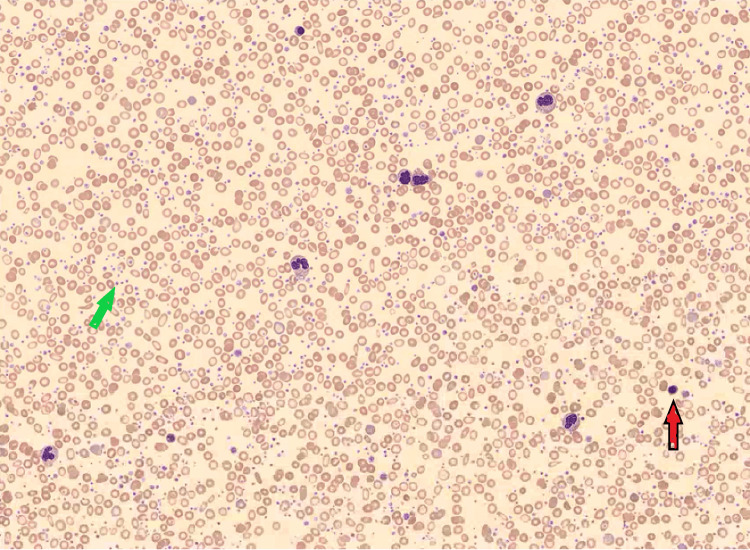
Peripheral smear showing a patient with an elevated white blood cell count, basophils (red arrow), and elevated platelet count (green arrow).

A 47-year-old woman was admitted to the hospital after experiencing occipital headaches and intermittent chest pain accompanied by dizziness, loss of appetite, and a feverish feeling for three consecutive days. Her medical history included a previous pregnancy with gestational hypertension, anemia, and heavy menstrual bleeding. Initial examinations, including an electrocardiogram (EKG), showed no signs of acute myocardial injury, and both the chest x-ray and CT head scan were normal. She was administered acetaminophen, which led to an improvement in her headache but caused her to develop pruritus. Laboratory tests revealed a white blood cell (WBC) count of 27,400 K/UL, with 12% basophilia and elevated myelocyte count and erythroblasts observed on the peripheral smear (Figure 1). Additionally, her hemoglobin (Hb) level was 7.0 g/dL, and her platelet (PLT) count was 2,515 K/UL as mentioned in Table 1. A follow-up platelet count showed 2,628 K/UL. Iron studies indicated a ferritin level of 4.9 ng/mL, iron level of 13 mcg/dL, total iron-binding capacity (TIBC) of 520 mcg/dL, and transferrin level of 371.5 mg/dL. Furthermore, her lactate dehydrogenase (LDH) was elevated at 393 U/L. Von Willebrand Factor (VWF) antigen and ristocetin cofactor levels were within the normal range, as described in Table 2.

**Table 1:** Complete blood count with differentials and coagulation profile aPTT: Activated partial thromboplastin time, INR: International normalized ratio

Laboratory Test	Result	Reference Range
Hemoglobin	7.0 g/dL	12.0 - 16.0 g/dL
Red Blood Cell	4.02 M/UL	4.2 - 5.4 U/ML
HCT	24.7%	37%-47%
MCV	61.4 FL	81.0-99.0 FL
RDW	25.7%	11.4%-16.4%
Reticulocytes	2%	0.5%-1.5%
Platelets	2628 K/UL	150-450 K/UL
White blood cell	27.4 K/UL	4.8-10.8 K/UL
Neutrophils	70%	50%-70%
Neutrophils band count	3%	0%-8%
Lymphocytes	4%	20%-40%
Monocyte	1%	2%-8%
Eosinophils	4%	0%-6%
Basophils	12%	0%-2%
Myelocyte count	5%	0%
PT	14.1 sec	10.2-14.2 sec
aPTT	31.1 sec	26.5-34.7 sec
INR	1.3	0.9-1.2 INR

**Table 2:** Anemia panel, von Willebrand factor antigen and ristocetin cofactor level TIBC: Total iron-binding capacity, LDH: Lactate Dehydrogenase, VWF: Von Willebrand Factor

Laboratory Testing	Result	Reference Range
Iron	13 mcg/dL	23-139 mcg/dL
Ferritin	4.9 ng/mL	3.6-78.0 ng/dL
TIBC	520 mcg/dL	279-449 mcg/dL
Transferrin	371.5 mg/dL	213-361 mg/dL
LDH	393 IU/L	108-199 IU/L
VWF Antigen	124.9	63%-170%
Ristocetin cofactor level	77	40.3%-163.4%

The complete blood count (CBC) was repeated the next day, revealing a hemoglobin (Hb) level of 6.3 g/dL. In response to this, the patient was given two units of packed red blood cells (pRBCs) and initiated intravenous iron therapy. Subsequently, the patient's hemoglobin remained stable after the transfusion, but a slight decline in platelet (PLT) count was observed (1,829-1,873-K/UL) following the administration of intravenous iron therapy. To address the platelet count issue, the patient was prescribed hydroxyurea (HU) to reduce platelet levels. However, after four days of treatment, the platelet counts showed little to no significant change. Platepharesis was not considered since Von Willebrand antigen and Ristocetin cofactor levels were within normal range.

Bone marrow aspirate and biopsy were conducted, and the initial findings ruled out a JAK2V617F point mutation. Within the mixed population of maturing myeloid cells, B cells, and T cells, a small myeloid blast population of 3% was observed, and no abnormal myeloid maturation was detected. Mild marrow fibrosis was also present. CD14+ monocytes accounted for less than 1% of total cells. A mild increase in CD34, CD117, CD13, CD33, and HLA-DR positive blast cells, comprising approximately 3% of the total cells, was noted. The B-cells (4% of the total) showed polytypic characteristics, and the T-cells (9% of the total) did not exhibit pan T-cell antigen deletion. The CD4 to CD8 ratio was approximately fourfold. Initially, the anemia and red blood cell indices, with a relatively maintained RBC count but markedly decreased MCV, raised suspicion of underlying thalassemia over iron deficiency anemia. However, the former was ruled out through capillary electrophoresis, which showed a normal hemoglobin pattern with microcytosis, so most likely etiology of microcytosis and iron deficiency anemia can be related to heavy menstrual bleeding.

Finally, the conclusive results from peripheral blood and bone marrow analysis revealed the presence of only the wild-type JAK2 sequence. It tested negative for the JAK2V617F point mutation, negative for JAK2 gene mutations in exons 12-15, negative for CALR gene mutation, and negative for MPL mutation. However, it tested positive for a BCR-ABL1 translocation, thereby confirming a diagnosis of chronic myeloid leukemia (CML) in conjunction with the patient's clinical presentation. The patient was scheduled to commence outpatient treatment with an oral tyrosine kinase inhibitor.

## Discussion

CML comprises approximately 15% of adult leukemias. It may be diagnosed at any age but the median age of diagnosis is 67 years. It stands out among other cancers as it can be recognized by a sole gene mutation: the BCR-ABL1 fusion gene. This gene arises from the Philadelphia chromosome and encodes the BCR-ABL1 protein. Unlike regular ABL1, this protein features an enzymatic domain with heightened and constant tyrosine kinase activity due to its fusion with BCR. This uncontrolled tyrosine kinase activity has been associated with the underlying mechanisms of CML [1].

Untreated, CML follows a biphasic or triphasic clinical pattern, advancing through a chronic phase, an accelerated phase, and ultimately a fatal blast crisis [5]. In the chronic phase, the peripheral blood smear displays leukocytosis, caused by various stages of maturation of granulocytes [6]. The number of blasts typically remains below 10%, and symptoms are generally mild. In the accelerated phase, the blast count ranges from 15% to 30%, with basophils constituting more than 20% of the blood cells. Thrombocytopenia may also be present, accompanied by additional symptoms like fever, reduced appetite, and weight loss. Occasionally, the disease may progress directly from the chronic phase to the blast crisis, particularly if it involves lymphoid blasts. During the blast crisis, blast cells exceed 20% and have spread beyond the bone marrow. This phase resembles acute leukemia, with symptoms akin to those observed in the accelerated phase.

Around 85% of patients, including this individual, receive their diagnosis during the chronic phase of the disease and subsequently progress to the accelerated and blast phases within three to five years. The diagnosis of CML is determined based on histopathologic findings in the peripheral blood and the presence of the Philadelphia chromosome in bone marrow cells [7]. Moreover, there are other factors, such as spleen size and platelet count, used for predicting prognosis, as observed in the Sokal score. In this case, the patient exhibited a high-risk Sokal score of 3.9 due to factors such as being 47 years old, having splenomegaly, severe thrombocytosis, and 3% peripheral blasts.

In some cases, thrombocythemia might be the sole peripheral blood abnormality during the chronic phase of CML. Even when isolated thrombocytosis is observed, it is essential to consider the possibility of CML. The patient in this case initially presented with severe thrombocytosis, but anemia, basophilia, and splenomegaly were also detected. Although elevated uric acid is a typical finding in CML, it was not present in this patient. Essential thrombocytosis could also be considered initially, but a microscopic examination of the smear may help differentiate between the two conditions, as CML shows small megakaryocytes with hypolobulated nuclei [7]. This case emphasizes the significance of considering CML when only thrombocytosis is evident during the presentation.

Patients with thrombocytosis, especially extreme thrombocytosis, have been observed to face a paradoxical risk of bleeding [8]. Typically, the bleeding in such cases manifests as mucocutaneous in nature. The increased bleeding risk is likely due to a combination of factors. In cases of clonal thrombocytosis, platelet function abnormalities are believed to play a significant role. Additionally, regardless of the cause of thrombocytosis, bleeding may occur due to acquired von Willebrand's syndrome (AVWS), resulting from the heightened adsorption of large VWF multimers by the abnormally elevated number of circulating platelets [8]. In patients experiencing thrombocytosis-associated bleeding, it is essential to test for AVWS to assess any decrease in large VWF multimers. Moreover, for patients with extreme thrombocytosis, evaluating AVWS can be integrated into the risk stratification process, particularly for otherwise low-risk patients, before commencing antiplatelet therapy.

## Conclusions

Extreme thrombocytosis is a condition that demands immediate attention and thorough evaluation. Our case underscores the crucial significance of arriving at an accurate diagnosis, a process that entails genetic testing of both peripheral blood and bone marrow. This step is essential to distinguish between essential thrombocytosis and CML, as it significantly impacts the choice of appropriate treatment. Furthermore, our case highlights the utmost importance of cautious patient selection for aspirin therapy, particularly in cases of extreme thrombocytosis, as this heightened level can lead to an increased risk of bleeding complications. Careful management and monitoring are paramount to ensure the best possible outcomes for patients with extreme thrombocytosis in the context of CML.
